# The first year of the COVID-19 pandemic in the ECOWAS region

**DOI:** 10.4314/gmj.v56i3s.8

**Published:** 2022-09

**Authors:** Serge MA Somda, Aristide R Bado, Abdourahmane Sow, Virgil K Lokossou, Sybil Ossei-A-Yeboah, Tome Ca, Nanlop Ogbureke, Stanley Okolo, Issiaka Sombie

**Affiliations:** 1 Department of Planning and Health Information, West African Health Organisation, Bobo-Dioulasso, Hauts-Bassins, Burkina Faso; 2 Department Public Health and Research, West African Health Organisation, Bobo-Dioulasso, Hauts-Bassins, Burkina Faso; 3 Regional Centre for Disease Surveillance and Control, West African Health Organisation, Bobo-Dioulasso, Hauts-Bassins, Burkina Faso; 4 General Directorate, West African Health Organisation, Bobo-Dioulasso, Hauts-Bassins, Burkina Faso

**Keywords:** ECOWAS region, COVID-19 pandemic, Incidence, Case fatality, epidemiology

## Abstract

**Objective:**

to analyse the pandemic after one year in terms of the evolution of morbidity and mortality and factors that may contribute to this evolution

**Design:**

This is a secondary analysis of data gathered to respond to the COVID-19 pandemic. The number of cases, incidence rate, cumulative incidence rate, number of deaths, case fatality rate and their trends were analysed during the first year of the pandemic. Testing and other public health measures were also described according to the information available.

**Settings:**

The 15 States members of the Economic Community of West African States (ECOWAS) were considered.

**Results:**

As of 31st March 2021, the ECOWAS region reported 429,760 COVID-19 cases and 5,620 deaths. In the first year, 1,110.75 persons were infected per million, while 1.31% of the confirmed patients died. The ECOWAS region represents 30% of the African population. One year after the start of COVID-19 in ECOWAS, this region reported 10% of the cases and 10% of the deaths in the continent. Cumulatively, the region has had two major epidemic waves; however, countries show different patterns. The case fatality rate presented a fast growth in the first months and then decreased to a plateau.

**Conclusion:**

We learn that the context of COVID-19 is specific to each country. This analysis shows the importance of better understanding each country's response. During this first year of the pandemic, the problem of variants of concern and the vaccination were not posed.

**Funding:**

The study was funded by the International Development Research Centre (IDRC) under CATALYSE project

## Introduction

Since the emergence of COVID-19 in November 2019, the disease has been declared a Public Health Emergency of International Concern by the World Health Organization (WHO) on 31 January 2020, and finally, a pandemic in March 2021.

In the ECOWAS region, the first case of COVID-19 was identified in Lagos (Nigeria) on 27 February 2020^1^, and throughout March 2020, all 15 countries in the region reported cases. The last country to report its first case was Sierra Leone on 31 March 2020.^2^ One year after the pandemic reached the region, the question of how the disease has evolved and the countries' response remains unanswered.

Several publications have attempted to provide answers at country,^3^,^4^ sub-regional^5^–^8^ and continental^9^–^11^ levels. These studies have shown several differences in morbidity, mortality, and response to the pandemic. Information on the demographic profile of cases and especially a comparative analysis of the evolution of the pandemic and mortality is limited. Also, most of the publications did not cover the entire first year. They did not focus solely on the 15 ECOWAS countries, which share a regional economic space with dynamic and functional institutions that allow frequent exchanges between countries and collegial and harmonised decision-making to deal with public health situations. This area also presents an economical, cultural, linguistic, demographic and health context that differs among countries.

This work was undertaken by the West African Health Organisation (WAHO) to analyse the evolution of morbidity and mortality of the pandemic in countries after the first year and assess factors that may contribute to this evolution. This will help the organisation for a better understanding of the dynamics of the pandemic at the regional level, similarities, and disparities among countries, to document critical steps of the evolution of the pandemic during year one in West Africa, and to provide insights, recommendations, and strategic directions for the pandemic response.

## Methods

The pandemic data was collected on the WAHO outbreaks platform in April 2021. Up-to-date information and analytic visualisations can be obtained from the following: https://data.wahooas.org/outbreaks/.

The platform is fed daily based on the countries' situation reports. The data used in these documents are those validated and shared by the respective Ministers of Health and contain information on the number of new cases, new deaths, and new discharged persons. They are available as epidemiological situation reports shared by email, social media, or official national platforms. According to the country's information, other variables, such as the number of tests performed are collected. However, in cases where information is not published, other data sources were crosschecked to obtain consistent information.

Information on public health and social measures (PHSMs) for each country is provided by WAHO staff. This person served as a liaison, collecting information and requests from the countries, and bringing them to the institution's discussion at a higher level. These resource persons routinely summarise all national responses to covid19 in the respective countries at the WAHO level for better supranational coordination. These data collected were triangulated with data gathered and made publicly available by the Organisation for Economic Cooperation and Development^12^ and Oxford University.^13^

For testing strategies, we calculated the testing ratio, representing the number of tests performed per million population.

This considers the country's total number of tests, including multiple tests for one person. The number of tests by the firmed case is also calculated. These are the main indicators monitored by the Africa Center for Disease Control (CDC)^9^, which considers that the optimal number of tests by case, as recommended by the WHO is between 10 and 30.^14^

The period used for data analysis was between the first confirmed case in the region (27th February 2020) to one year after the last ECOWAS country confirmed its first case (31st March 2021). Descriptive analyses were done on key indicators. The daily incidence rate was calculated as the number of new cases per million population. Cumulative incidence rates were calculated as the number of total cases by one million population. Case fatality rates (CFR) were calculated as the number of deaths reported per 100 confirmed cases. Trend analyses were performed. The daily information was smoothed using a general additive model (GAM) smoother.^15^

The effective reproductive number is defined as a time function and the^16^ The *EpiEstim* R package^17^,^18^ was used to estimate the parameters. This uses a two-step procedure to estimate the effective reproduction number from data informing the serial interval and from data on the incidence of cases over time.^19^ The confirmation dates were used for the estimation as the date of infection could not be obtained. The serial interval was simulated as an undefined distribution with an expectation of the mean of 3.6 days and an expectation of the standard deviation of 3 days. The serial interval and the reproductive number are simulated using a Monte Carlo approach.

## Results

### Total number of cases and cumulative incidence

Table 1 presents a summary of the key indicators for all countries one year after the first case was confirmed in Sierra Leone. At the end of year one of COVID-19, the region reported 429,760 confirmed cases from the 15 ECOWAS member States, with 5,620 deaths. Nigeria, followed by Ghana, Côte d'Ivoire, Senegal, and Guinea, were the countries where most cases were reported. These countries reported more than ¾ of the cases in the region.

**Table 1:** Summary of the COVID-19 pandemic situation in the ECOWAS region after one year (March 2020 – March 2021)

Country	Date 1^st^ Case	Population^1^	Confirmed Cases	Cumulative Incidence Rate (per 1 million)	Deaths	CFR (%)
**Ecowas Region**	**02/27/2020**	**386,908,402**	**429,760**	**1,110.75**	**5,620**	**1.31**
**Nigeria**	02/27/2020	200,963,599	160,493	798.62	2,012	1.25
**Senegal**	03/02/2020	16,296,364	38,399	2,356.29	1,032	2.69
**Togo**	03/06/2020	8,082,366	10,249	1,268.07	109	1.06
**Burkina Faso**	03/09/2020	20,321,378	12,748	627.32	149	1.17
**Cote d'Ivoire**	03/11/2020	25,716,544	43,542	1,693.15	242	0.56
**Ghana**	03/12/2020	30,417,856	89,893	2,955.27	734	0.82
**Guinee**	03/12/2020	12,771,246	20,083	1,572.52	127	0.63
**Benin**	03/16/2020	11,801,151	7,100	601.64	90	1.27
**The Gambia**	03/17/2020	2,347,706	5,485	2,336.32	165	3.01
**Cabo Verde**	03/20/2020	549,935	17,121	31,132.77	165	0.96
**Niger**	03/20/2020	23,310,715	5,003	214.62	186	3.72
**Guine Bissau**	03/24/2020	1,920,922	3,634	1,891.80	61	1.68
**Liberia**	03/24/2020	4,937,374	2,042	413.58	85	4.16
**Mali**	03/24/2020	19,658,031	9,998	508.60	384	3.84
**Sierra Leone**	03/31/2020	7,813,215	3,970	508.11	79	1.99

1 2019 population from World Bank estimation: https://data.worldbank.org/indicator/SP.POP.TOTL

The cumulative incidence showed that the region counted 1,111 cases per million inhabitants after one year of the epidemic. There were differences among countries. Cabo Verde presents a high incidence, and Niger, low values. Eight countries (Cabo Verde, Ghana, Gambia, Senegal, Guinea Bissau, Côte d'Ivoire, Guinea, and Togo) had an incidence of over 1,000 cases per million.

### COVID-19 incidence cases during the first year

**Figure 1:**
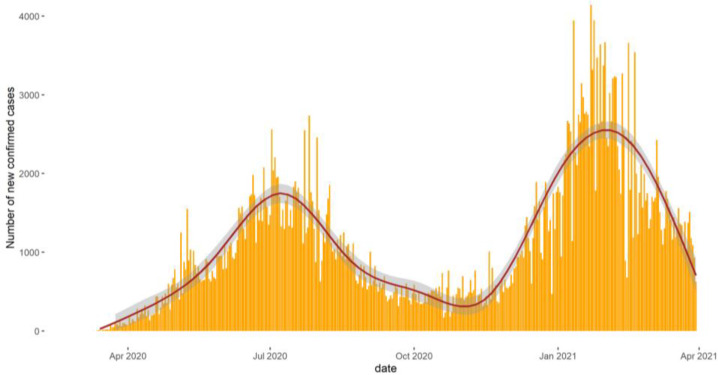
Trend of the number of daily new cases of COVID-19 in Ecowas member States

Figure 1 shows the trend of the new daily cases within the entire ECOWAS region from February 2020 to March 2021. Two epidemic waves can be noticed. The first one occurred between April and October 2020 with a peak observed in early July 2020. The second wave, which was shorter than the first one, started at the end of November 2020 with a peak around February 2021, and was more significant in terms of the number of daily cases.

**Figure 2:**
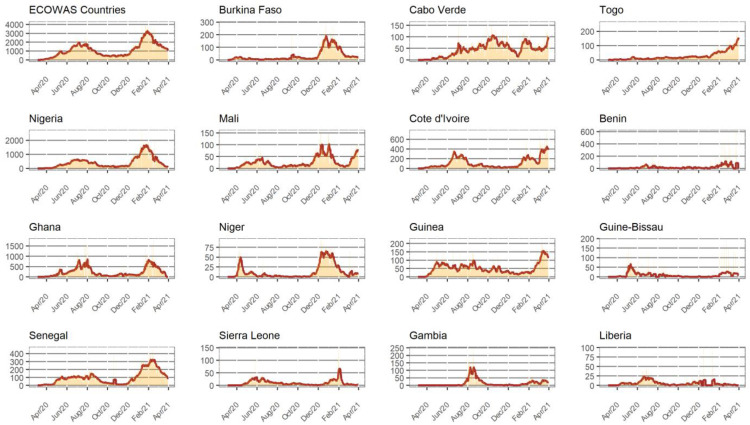
Evolution of the number of COVID-19 confirmed cases during the first year of the pandemic by country

Figure 2 presents the evolution of new daily COVID-19 cases for each of the 15 ECOWAS countries. Four groups can be observed. The first group of countries have the regional pattern. These countries generally present a larger number of cases and influence the pooled data. The pattern is the two major waves, the first presenting a peak around July 2020 and the second one in February 2021. Nigeria, Ghana, and Senegal are the main representatives of this group. The second group presents more than two peaks. For Burkina Faso, two small waves were observed in May and October 2020 before the larger one in February 2021. Mali, Niger, and Sierra Leone manifested this specific pattern with a starting third wave. The third group includes countries like Cabo Verde and Cote d'Ivoire, the first wave didn't really end before the occurrence of the next one. This means that as the transmission starts decreasing, another wave occurs, and the curve started increasing again. Finally, for the last group of countries like Guinea-Bissau, Togo, and Benin no epidemic waves were observed.

### COVID-19 deaths during the first year

**Figure 3:**
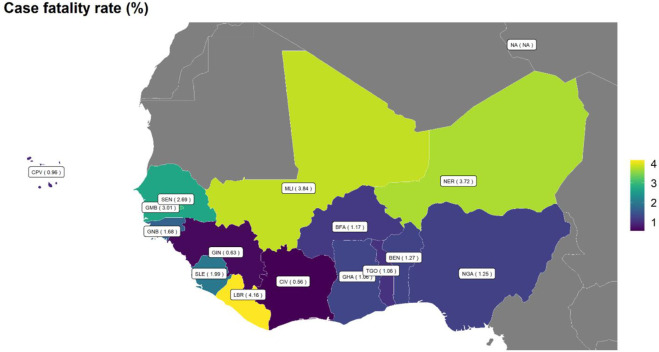
COVID-19 case fatality rate (%) by country in the ECOWAS region from March 1^st^, 2020, to March 31^st^ 2021

One year after the epidemic in the ECOWAS region, 1.31% of all confirmed cases died. The case fatality ratio was quite different from one country to another (Figure 3). The lowest fatality rates are observed in Côte d'Ivoire, Guinea, Ghana, and Cabo Verde. The highest case fatality rate was in Liberia (4.16%).

**Figure 4:**
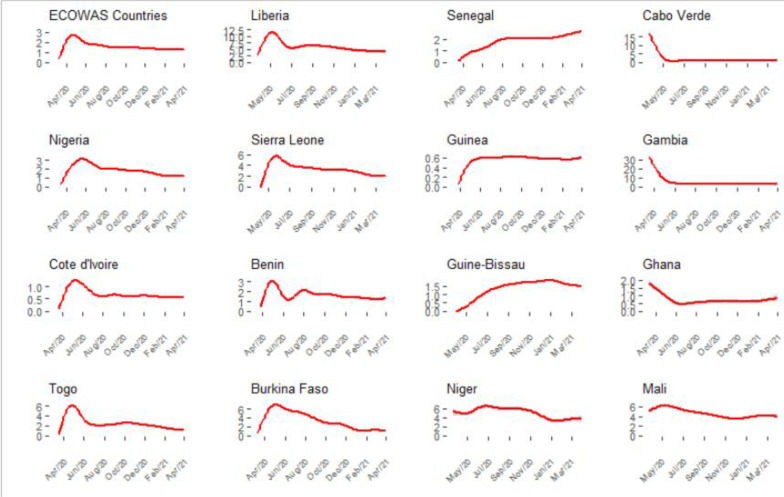
Evolution of COVID-19 case fatality rates during the first year of the pandemic in ECOWAS member states

The trend of the case fatality ratio showed a fast growth between March and April. In May and June, it was nearly 3% of the confirmed cases (more than 6% in Togo, Mali, Liberia, Burkina Faso). It decreases to a plateau value from August and September which corresponds to the value represented in Figure 4. Some countries show steady trends. We have the example of Guinea-Bissau and Senegal. The curve in Guinea-Bissau seems to have reached a peak in January, this is not the case in Senegal where the case fatality is steadily increasing with the time.

**Figure 5:**
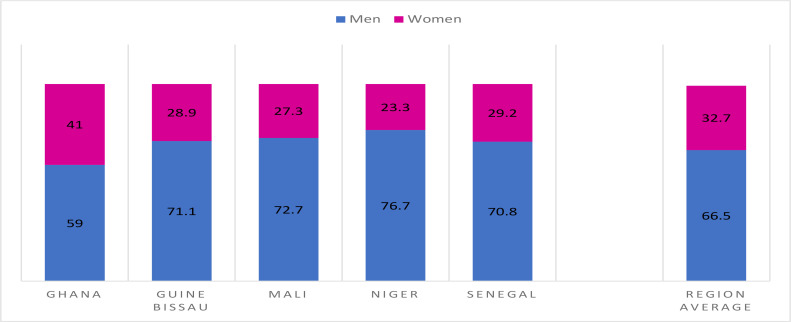
Gender distribution of COVID-19 deaths for some countries (%)

Data about the characteristics of the deaths are scarce. It was however possible to gather the main comorbidities in February 2021. Some countries could provide the distribution of deaths by gender. For all of them, higher percentages of deaths were male. When the data are pooled, we observe that 2/3 of the deaths in most countries are males (Figure 5).

**Figure 6:**
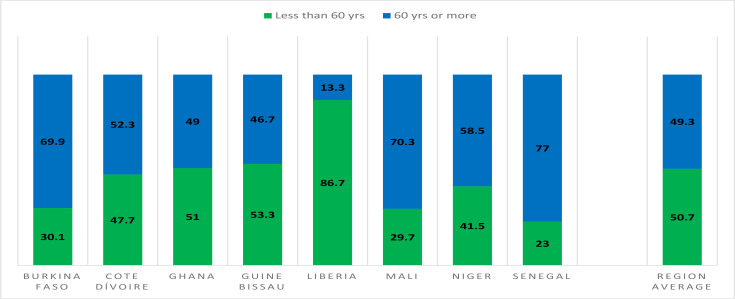
Age distribution of COVID-19 cases in some countries ECOWAS member states from March 1^st^, 2020, to March 31^st^ 2021

In terms of age distribution, more countries are collecting this information. However, the definition of the thresholds for organising the age distribution varies from one country to another, which makes them difficult to compare. Figure 6 shows a simplified breakdown (over or under 60 years) of a threshold that was common to all countries. In terms of results, even though the proportion of persons over 60 years in the population is very low, they represent almost half of the deaths. However, in Liberia, the reported deaths were generally younger as only 13% were over 60 years (Figure 6).

Table 2 presents the distribution of main comorbidities in some ECOWAS Member States. The most reported comorbidity was heart diseases which represents almost half of the available information. Diabetes was also frequent and represents around ¼ of the cases. Several cases of renal diseases and cancers were also observed. It is important to add that most of the cases with morbidities had multiple comorbidities.

**Table 2:** Distribution of main comorbidities associated with COVID-19 deaths (%) in some ECOWAS member States from March 1^st^, 2020, to March 31^st^, 2021

	Deaths	Heart Disease	Renal Disease	Diabetes	Cancer	Other
**Burkina Faso**	125	65.6	7.2	24.0	-	3.2
**Cabo Verde**	135	30.9	10.1	17.4	2.7	38.9
**The Gambia**	11	9.1	-	81.8	9.1	-
**Ghana**	358	51.7	3.7	40.6	4.0	-
**Guine Bissau**	45	44.4	-	17.8	-	37.8
**Mali**	204	43.9	1.5	37.3	-	17.3
**Niger**	160	50.0	1.3	23.8	1.9	23.1
**Senegal**	689	41.7	4.5	19.6	4.1	30.2
**Sierra Leone**	79	25.3	3.6	18.7	1.8	50.6
**Overall**	1806	43.6	4.1	26.4	2.7	23.1

### Effective reproductive number

**Figure 7:**
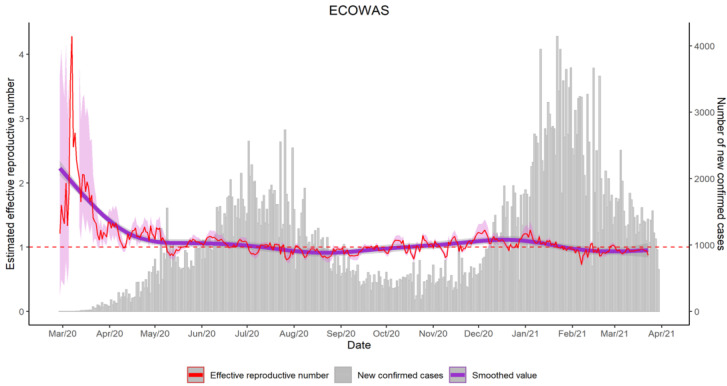
Regional estimation of the effective reproductive number during the first year of COVID-19 in the ECOWAS region

The effective reproductive number is a predictive tool to monitor the evolution of the epidemic. Figure 7 shows that the maximum level of this indicator was 4, at the early periods of the outbreak. Then, the number stabilised between 0.5 and 1.5, showing slow increases or slow decreases during the different waves.

Looking at the trends, the reproductive number remained more than one until July 2020; thus, predicting the first epidemic peak. Then, number stayed below the reference value of one for three months. In November, it passed the threshold, showing that the epidemic restarted a new wave; after which, there was an approximately constant trend.

**Figure 8:**
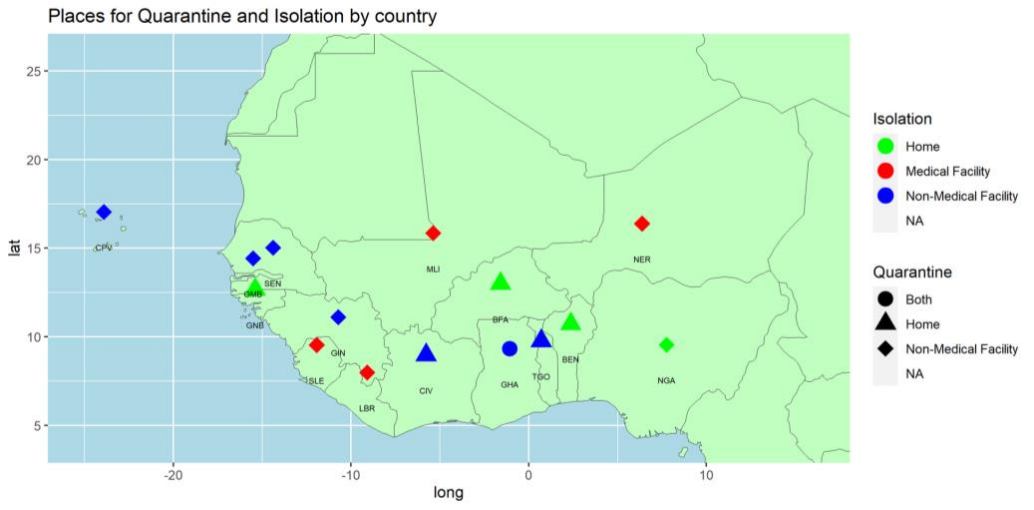
Mode of quarantine for contacts and travellers and mode of isolation of asymptomatic cases by country

Regarding the need for quarantine concerning contacts and travellers, they were either in non-medical facilities like hotels or at home. Nine-member States were keeping all the cases in non-medical facilities. Ghana adopted both strategies, depending on the case. Figure 8 presents where people were isolated or quarantined when they were positive or suspected.

### Case detection and testing

Table 3 presents the number of tests by population and the number of cases by tests on 6th April 2021. In the ECOWAS region, 5.9 million tests were performed during the first year of the pandemic. The number of tests per million population was 15,390. We observe differences between countries. Cabo Verde recorded the highest testing ratio per population, followed by Benin, Togo, Ghana, and Guinea-Bissau. According to the number of cases by test, Cabo Verde presented the lowest number of cases by test. The ECOWAS region conforms to the WHO's recommendation of ten to 30 tests per reported COVID-19 case as a measure for optimal testing coverage in the population. However, Cabo Verde is below the recommendation threshold while Liberia and Benin exceeded the value.

**Table 3:** Number of tests, positivity rate and number of tests per population in the ECOWAS countries

	Population	Tests	Cases	Testing Ratio	Tests / Cases
**ECOWAS Region**	**386,908,402**	**5,954,507**	**439,664**	**15,390**	**14**
**Cabo Verde**	549,935	130,599	18,023	237,481	7
**Benin**	11,801,151	548,484	7,515	46,477	73
**Togo**	8,082,366	283,560	11,310	35,084	25
**Ghana**	30,417,856	1,016,808	91,009	33,428	11
**Guinea Bissau**	1,920,922	59,431	3,662	30,939	16
**Senegal**	16,296,364	447,147	39,127	27,438	11
**Guinea**	12,771,246	335,390	20,510	26,261	16
**The Gambia**	2,347,706	58,454	5,564	24,898	11
**Cote d'Ivoire**	25,716,544	539,507	44,854	20,979	12
**Sierra Leone**	7,813,215	140,170	3,990	17,940	35
**Liberia**	4,937,374	85,675	2,061	17,352	42
**Mali**	19,658,031	238,212	10,758	12,118	22
**Nigeria**	200,963,599	1,803,177	163,388	8,973	11
**Burkina Faso**	20,321,378	175,907	12,845	8,656	14
**Niger**	23,310,715	91,986	5,048	3,946	18

### Public health and non-pharmaceutic response to the pandemic

Table 4 provides a summary of the different actions by the governments. We limited the list to the most visible actions that affect more the populations. Thus, other actions like the organisation of the response teams, the development of guidelines, training, implementation of actions, etc. are not listed here. The first public health and non-pharmaceutic measure to mitigate the spread of the disease was the management of the cases. All symptomatic cases were managed in dedicated health facilities with the required isolation facilities. Three approaches were observed regarding asymptomatic cases isolation. Many countries (Cabo Verde, Cote d'Ivoire, The Gambia, Guinea, Senegal, and Togo) preferred isolating the patients in non-medical facilities like hotels. Four-member States (Liberia, Mali, Niger, and Sierra Leone) were isolating all their cases in medical facilities while four (Benin, Burkina Faso, Guinea-Bissau, and Nigeria) were asking the asymptomatic cases to self-isolate at home.

**Table 4:** Countries non-pharmaceutical health measures undertaken to control COVID-19 expansion region

PHSM	Partial Lockdown	Curfew	Closure/Restriction International Borders	Gathering Forbidden	Schools and University Closure	Closure of Restaurants, Café, Night Clubs, ...
**Bénin**	30-March		18- March		30- March	-
**Burkina Faso**	26-march - 4-may (if < un positive case)	21- March	21- March	20- March	14- March -	20- March
**Cabo Verde**	20- March			16- March		18- March
**Cote d'Ivoire**	23- March	23-march - 7-may & 14-may	20- March	16- March - 7-may	16- March -	16- March - 7-may & 14-may
**The Gambia**			23- March	17- March		27- March
**Ghana**	30-march - 20-april		22- March	15- March	17- March -	
**Guinea**		31-march - 15- may (except Conakry region)	26- March	19- March	25- March -	26- March
**Guinea-Bissau**		27-Apr	18- March	16- March		16- March
**Liberia**	10-Apr		31- March	16- March		21- March
**Mali**		26-march - 8- may	25- March	17- March	19- March -	17- March
**Niger**	29- March	27- March	17- March	17- March	17- March -	18- March
**Nigeria**	30-march - 21-may					
**Senegal**		23- March	19- March	15- March	14- March -	
**Sierra Leone**		9-Apr	27- March	18- March		
**Togo**	21- March	2-Apr	20- March	16- March		20- March

With regard to the political measures to respond to the epidemic, almost all countries promoted community social distancing. These were organised in terms of large communication actions, cancellation, postponement, or modification of certain gathering activities, etc. Seven ECOWAS member states had a period with night curfews (Burkina Faso, Guinea-Bissau, Guinea, Nigeria, Senegal, Sierra Leone, and Togo). Eight countries had partial lockdown, but no complete lockdown was observed in any ECOWAS member state.

One important action taken by governments is the border closure. All the ECOWAS countries (except Benin) closed their borders for a long period of time. After setting up measures in the airports, the air borders were opened in August 2020. The land borders are still closed as setting up all the safety and control protocols are yet to finish.

## Discussion

After one year of the pandemic, 429,760 persons were confirmed COVID-19 in ECOWAS Member States with 5,620 deaths. ECOWAS region represents around 30% of the African population. However, both the confirmed cases and the deaths from the region represent 10% of the African cases and deaths. The rapid closure of borders and the implementation of non-pharmaceutical measures may explain the apparent containment of the pandemic. At this moment, Africa accounts for 2% of the COVID-19 cumulative cases and 3% of the deaths globally.^20^ While Africa was declaring 3,061,438 cases, the Americas were counting 55,243,776 and Europe 44,191,579 with 126,372,442 cases declared globally.^20^ In Africa, data from equivalent period from the Southern WHO African region had the highest number of cases and deaths.^21^ These are followed respectively by the Northern region, the Eastern region, the Western region and the Central region. The Western region in Africa is finally the fourth in the continent both in terms of cases and deaths and has the highest recovery rate in the continent.^21^ Some factors may help to explain these differences between regions in Africa. These factors include climate, population size, population density, implementation of and compliance with non-pharmaceutical public health measures, and screening capacity. The potential implications of these factors were discussed by Cabore et al.^10^ in 2020. Obande et al.^21^ also recognised that even though these factors are of interest, they could not be analysed separately. The effect of these factors on the epidemiological profile of the disease was finally high-lighted by Gesesew et al. in a systematic review.^22^ For example, they found that COVID-19 infection and related deaths were affected by internal governance (including political democracy and economic policy).

The countries' contribution to the pandemic was not proportional to their population. For example, Nigeria, which has the largest number of cases, representing 52% of the regional population, contributed only 37% of the cases. However, Cabo Verde and Ghana, representing less than 1% and 8% of the regional population respectively, contributed 4% and 21% of confirmed cases in the ECOWAS region. This can be due to the level of preparedness of the countries to address such an epidemic and also to the disparities in terms of decision taken by the different governments. Lone and Ahmad^23^ described this outbreak as a “wake-call for Africa”.

Then, several actions are ongoing to improve the capacity of the countries in terms of preparedness for response to health emergency. International bodies should also reinforce their governance to be able to better coordinate actions and decisions at regional level when the emergency come to concern more than one state.

Countries with a trend similar to the regional trend were the largest contributors to the pandemic at the regional level. Nigeria, Ghana, Côte d'Ivoire, and Senegal alone accounted for 77% of the region's cases. Trends in Benin and Guinea-Bissau are very difficult to analyse as the data were more usually back log information for long periods of time. The information is transmitted irregularly and with data grouped over several days.

Important comments are made regarding the published statistics on the pandemic, especially in developing countries. In fact, the number of confirmed cases will depend on the testing strategy. Many cases are asymptomatic. As an example, Fergusson et al.^24^ reported that the data from China and repatriating flights suggests that 40% – 50% of infections were not detected as “cases”. Thus, when the strategy consists of testing people showing symptoms or people indicated as known contacts (under certain conditions), many cases will be lost. Not yet published sero-prevalence surveys that were run in the region should confirm the large number of missed COVID-19 cases.^25^ Some experienced severe COVID-19 episodes without being diagnosed. Also, the number of deaths from the disease is probably underestimated.

The incidence curves are identical to those for cases per day. However, when comparing the levels, Cabo Verde has a very high incidence compared to the other countries. This country, with the smallest population, has many cases comparable to the other countries. However, looking at the number of tests per population (testing ratio), Cabo Verde has tested the equivalent of more than a quarter of its population during the year. However, if we look at the number of tests required for a confirmed case, the country has the lowest value. It is below the thresholds recommended by WHO and Africa CDC.^9^ In this sense, one could question the quality of the strategy in place in Cabo Verde. However, Benin and Liberia reported very high test per case ratio. The situation in Benin requires further study. This could be due to the strict regulations for international travellers in this country since the outbreak of the disease. Liberia was systematically testing dead bodies in the morgue at the beginning of the epidemic. This potentially explains both the case fatality and this indicator.

The fact that most countries are within the test per cases recommended range could be explained by the improvement in the countries' testing capacities. For example, from February to September 2020 the region went from 2 to more than 200 laboratories offering COVID-19 testing capacity.^26^ In January, Senegal's Institute Pasteur was the only referral laboratory in the region, responsible for testing samples from other countries.^27^ However, after the first wave of the pandemic, countries experienced a relaxation of measures and surveillance. This was due to fatigue, devolution processes of care functions, etc.^28^ The challenge of having a good testing strategy in Africa is important. One of the main aspects of this challenge is that Africa is dependent on external suppliers.^29^ Despite the pooled procurement of tests facilitated by WHO global access to COVID-19 tools,^30^ the continent remains underserved in terms of material and reagents.

The Liberian strategy of testing dead bodies was a way to adjust the mortality indicator which was described as wrong.^31^ When the civil registration is working, the excess mortality can be estimated. This approach is currently used by many researchers to model the epidemic.^32^ Testing dead bodies could help estimate this excess mortality.^33^ However extensive statistical estimation has to be done on this aspect. As already stated, Liberia had the highest case-fatality rate because at the beginning of the epidemic all dead bodies were systematically tested. It is followed by Mali, Niger, and The Gambia. In these countries, the quality of care could explain the values obtained. The rapid rise in the first two months of the pandemic and the regression of the regional case fatality curve could be explained by the quality of care at the very beginning of the pandemic, when the countries had neither the structures nor the equipment nor the adapted protocols. This then fell and generally stabilised around an equilibrium point. Yet, three countries, Guinea-Bissau, Guinea, and Senegal, showed increasing lethality curves. The situation in Senegal could be explained by the delay in diagnosis and the refusal of patients to adhere to the protocol, due to the stigmatisation of cases and finally the overflow of care infrastructures. The second wave in Senegal concerned more elderly people, which impacts on lethality.^34^ For Guinea-Bissau and Guinea, this could be the effect of the weakness of the health system, which needs more support to respond to this disease while many other health problems are emerging. Finally, it is important to study the effect of co-morbidities on deaths. The main limitation of the study is the quality of the data. Data were collected from official country reports. These do not present information on cases such as age, sex, co-morbidities, etc. Moreover, these inputs are not regular and often include carryovers to previous periods. The sparse data on co-morbidities show how important it is to monitor this aspect. Anjorin et al.^35^ have identified obesity, cardiovascular diseases, diabetes, and other viral respiratory diseases as worsening COVID-19 prognosis. These have been found among the few reported data on deaths. For tests, data are often not available. It is also difficult to discuss strategies based on the figures obtained. Researchers attempted to prepare databases with robust information.^36^ Unfortunately, these lack exhaustivity and generally, the data from some ECOWAS countries are usually missing. Finally, the information needed to explain the situation was generally lacking as systematic event recording was not done in the countries.

This analysis shows the importance of a better understanding of the response in each country. This confirmed what was alleged before.^37^ The first year of the epidemic, the problematic of variants of concern was not posed. The first variants of concerns appeared in the region on February 14th.^38^ This did not really influence the course of the pandemic in the first year. Additionally, the country responses were more based on non-pharmaceutical public health measures improvement of detection and improvement of the care settings. The problem of vaccination appeared later. The vaccination was launched in the first country in the region on March 5^th^ 2021.^39^

All this information confirms that the pattern which was observed was principally influenced by the countries resilience and their capacities to react to new emergencies instead of important changes in the structure of the pandemic. The non-pharmaceutic measures and the adherence from the population was key to inflate the epidemic curve. The rapid relaxation in both political and public attention was also a key point in the resurgence of the disease and the appearance of the second wave.

The importance of the case fatality rate at the beginning of the pandemic shows that almost none of the countries was really prepared for the disease at the beginning. Some basic procedures were generally not respected due to the multiplicity of instructions from several sources or due to the fear of being contaminated or the fear of being sanctioned in the event of an imprudent action.

Subsequent studies will focus on the actions of each country and the implications of these on the control of the pandemic. Studies targeting recent period of the pandemic should necessarily consider the new changes observed in the pandemic. The first major change is the emergence of variants of concerns. These variants bring changes in the infectiousness of the disease and also the fatality.^40^ Some variants are more likely to infect the categories of people who used to be at low risk. These should also consider the introduction of the immunisation. The vaccination brings an important new weapon to fight against the pandemic. This is supposed to flatten the infection curves and to reduce the case fatality. All the ECOWAS countries are now in massive vaccination policies while they are still promoting non-pharmaceutical measures like physical distancing, mask wearing, and other preventive measures.

## Conclusion

At the end of the study, we learn that the context of COVID-19 is specific to each country. The evolution of the epidemic is strongly influenced by the country context.
